# Urticaria multiforme-like: a COVID-19 infection

**DOI:** 10.11604/pamj.2020.37.286.26740

**Published:** 2020-11-30

**Authors:** Cristiana Canelas Mendes, Rita Pimenta

**Affiliations:** 1 Department of Internal Medicine 2, Centro Hospitalar Universitário de Lisboa Norte, Hospital de Santa Maria, Lisboa, Portugal,; 2 Department of Dermatology, Centro Hospitalar Universitário de Lisboa Norte, Hospital de Santa Maria, Lisboa, Portugal

**Keywords:** COVID-19, cutaneous manifestations, skin

## Image in medicine

An 18-year-old, otherwise healthy woman, presented to the emergency department with a three-day history of acute-onset diffuse rash. She denied recent introduction of new drugs or dermocosmetic products and previously adverse drug reactions or allergies. She hadn't had any known contact with anyone with confirmed SARS-CoV-2 infection. On examination, she had a temperature of 38.6°C. Physical examination showed a disseminated, asymptomatic and not evanescent annular erythematous urticarial papules and plaques, some with targetoid appearance, mostly located at the back (A), chest (B), inguinal zone (C), distal portions of upper (D) and lower limbs and forehead. The palmoplantar skin and mucous membranes were spared. Laboratory tests showed d-dimer elevation (0.43 μg/ml, normal range 0.0-0.25 μg/ml). Chest radiography and computed tomographic pulmonary angiography was normal. A reverse-transcriptase-polymerase-chain-reaction test for severe acute respiratory syndrome coronavirus 2, performed on a nasopharyngeal swab, was positive. A diagnosis of COVID-19 infection was made. On the sixth day of follow-up, fevers and rash were disappeared. The particular interest of this case is the inaugural appearance of a cutaneous manifestation, before any other symptom. It is essential to take into account and promote the recognition among clinicians of this possible skin manifestation of COVID-19, because an early diagnosis, could contribute to break the chain of transmission immediately.

**Figure 1:**
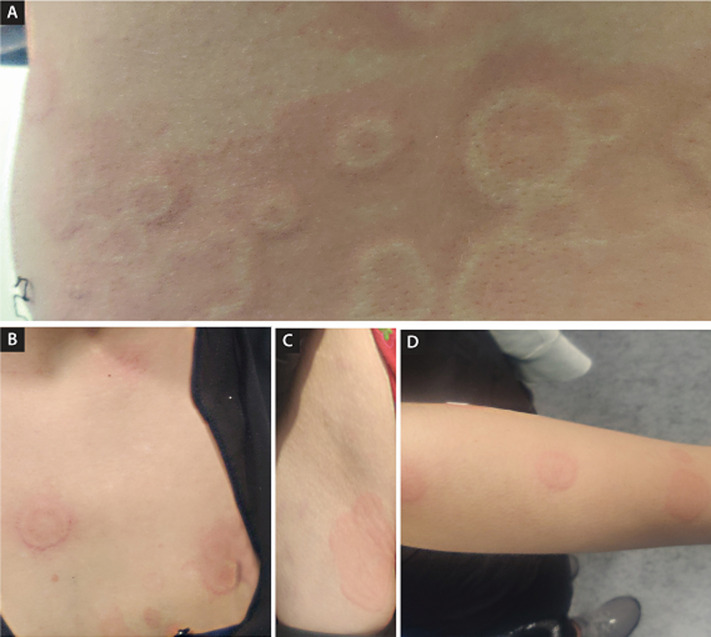
urticaria multiforme-like: a COVID-19 infection

